# Disseminated adenovirus infection in a patient with a hematologic malignancy: a case report and literature review

**DOI:** 10.2144/fsoa-2019-0072

**Published:** 2019-08-28

**Authors:** Akane Takamatsu, Yasuaki Tagashira, Shinya Hasegawa, Hitoshi Honda

**Affiliations:** 1 Division of Infectious Diseases, Tokyo Metropolitan Tama Medical Center, Tokyo, Japan

**Keywords:** disseminated adenovirus infection, hematologic malignancy, hematopoietic stem cell transplantation, human adenovirus, viremia

## Abstract

Human adenoviruses cause a wide spectrum of illnesses, including invasive infections, in immunocompromised hosts. We report a case of disseminated adenovirus infection following unrelated cord–blood transplantation in a 46-year-old male with a lymphoma. A review of the literature on disseminated adenovirus infections in adult patients with hematopoietic stem cell transplantation has also been included. Despite antiviral therapy, the mortality rate in hematopoietic stem cell transplantation recipients with a disseminated adenovirus infection is as high as 72%, and estimating the risk of human adenovirus infection in a timely manner is crucial to improving outcomes.

## Lay abstract

Human adenoviruses are common viruses that cause a range of illness such as bronchitis, gastroenteritis and conjunctivitis. We recently experienced a case of disseminated adenovirus infection following hematopoietic stem cell transplantation in a patient with a lymphoma who received chemotherapy. Although disseminated adenovirus infection is not a rare disease in immunocompromised patients, it is noteworthy that its mortality remains high and it may not frequently occur. Therefore, this work provides a case presentation and a review of disseminated adenovirus infection in patients with hematopoietic stem cell transplantation in order to aid our understanding of the nature of illness of human adenovirus infections in immunocompromised patients.

Human adenoviruses (HAdVs) are nonenveloped, double-stranded DNA viruses. There are seven species (A–G) comprising 90 types that can affect different organs and cause a wide spectrum of diseases in both immunocompetent and immunocompromised hosts [1,2]. Although the clinical course is usually mild and self-limiting, HAdV infections can cause lethal outcomes in immunocompromised patients. Herein we report a case of disseminated adenovirus infection in an adult patient with a lymphoma who underwent unrelated cord–blood transplantation (CBT). A review of the current literature on this topic has also been included.

## Case presentation

A 46-year-old Asian male with a preliminary diagnosis of Burkitt lymphoma was treated with hyper-CVAD/MA (cyclophosphamide, doxorubicin, vincristine, dexamethasone, methotrexate and cytarabine) and rituximab. Subsequently, he received the definitive diagnosis of double-hit lymphoma, and treatment with IVAC (ifosfamide, etoposide and cytarabine) and rituximab was begun in 2017. Despite this treatment, the lymphoma progressed, and the patient was transferred to our hospital in January 2018. He was first treated with DeVIC (carboplatin, etoposide, ifosfamide and dexamethasone) and rituximab but showed no response and underwent unrelated CBT on February 2018 following a myeloablative conditioning regimen. A fever developed on day 2 after CBT, and a course of empiric antimicrobials (cefepime, vancomycin and metronidazole) was started. On day 11 of CBT, an intermittent dry cough with persistent fever and mild rhinorrhea developed. Computed tomography of the chest revealed bilateral interstitial infiltrates in his bilateral lungs, and foscarnet and atovaquone were started based on a presumptive diagnosis of cytomegalovirus (CMV) infection and pneumocystis pneumonia. On day 14, HAdV was detected in a sputum sample, and his serum HAdV viral load was 3.2 × 10^7^ copies/ml. CMV-PCR was negative both in the sputum and blood. In addition, *Pneumocystis jirovecii* and other respiratory viruses were not detected by PCR analysis of the sputum. The blood and sputum cultures were also negative. Foscarnet, atovaquone and metronidazole were stopped, and he received one dose of cidofovir on day 15. Cefepime and vancomycin were continued for his febrile neutropenia. However, cidofovir was unable to be continued due to his deteriorating renal function. On day 19, he experienced a seizure, and his cerebrospinal fluid was positive for HAdV. He died on day 23 from multiorgan system failure.

## Discussion

HAdV is one of the commonest causes of respiratory tract infections in children [1] and is responsible for up to 2% of adult cases of community-acquired pneumonia [3]. Moreover, HAdVs are increasingly being recognized as the causative pathogens in infections in immunocompromised hosts; however, HAdVs continue to provide clinical challenges both in diagnosis and treatment. Lymphocytes are crucial for controlling viral infections, including HAdV [1,4]. The risk factors for disseminated adenovirus infections include allogeneic stem cell transplantation from a haploidentical donor or unrelated cord–blood graft and any immunosuppression with T-cell depletion [1].

To investigate the clinical manifestations and prognosis in immunocompromised patients with a disseminated adenovirus infection, we searched the PubMed database for previous (up to July 2018) literature on disseminated adenovirus infections in adult patients older than 19 years of age with a hematologic malignancy. The search terms used were ‘adenoviridae infections’ (Mesh) AND (‘humans’ [MeSH Terms] AND English [lang]) AND (‘viremia’ [Mesh] OR ‘disseminated’ [All Fields]). A total of 190 articles were found, and all the titles and abstracts of potentially relevant studies were vetted. Full-text articles were reviewed as needed. Articles were excluded for any of the following reasons: patients’ age <20 years; unavailability of articles; insufficient or missing patient data; or absence of hematopoietic stem cell transplantation (HSCT). In total, 129 cases in 26 articles were finally included, with the present case being the 130th. These cases are summarized in Supplementary Table 1, and a complete list of clinical characteristics of the patients included in the review is shown in Supplementary Table 2 [5–30]. In summary, the average patient age was 39.5 years, 75/106 (70.8%) cases occurred in males, and almost all the cases (128/129 cases) involved an allograft HSCT. Acute graft-versus-host disease occurred in 66/100 (66%) patients, and co-infection due to other viruses was observed in 27/51 (52.9%) patients. The timing of the infection after HSCT varied (range: -4 days to >6 years). At least 46/93 (49.5%) of the cases were associated with a pulmonary infection, 80/105 (76.2%) patients received an antiviral agent for the HAdV infection, and 94/130 (72.3%) patients died. Time from the diagnosis of HAdV infection as confirmed by the onset of the symptoms or the first adenovirus-positive results of PCR analysis of the patients’ blood to treatment with antiviral agents or death ranged from 0 to 65 days and 2 to 116 days, respectively.

The clinical manifestations of HAdV infection can range from mild respiratory or gastrointestinal symptoms to severe hemorrhagic enteritis, cystitis, pneumonia, hepatitis or encephalitis. Occasionally, concomitant involvement of several organs, which is associated with increased morbidity and mortality due to multiorgan failure, is encountered [1]. Due to the variety of symptoms, performing a diagnostic test in a timely manner is often challenging. Although a previous study indicated that routine monitoring for viremia by PCR was beneficial in pediatric HSCT recipients [31], a pre-emptive therapy for viremia alone has not been established due to the toxicity of commonly used antiviral agents in adult HSCT recipients [32]. Moreover, HAdV infections can be acquired *de novo* or via reactivation, and recipient and/or donor serostatus may have no impact on the occurrence of these infections [33]; thus, the best timing for testing is unclear despite the clear presence of certain risk factors. In our review, almost half the cases had pneumonia, followed by colitis and urinary tract infection. In these situations, it may be useful to consider HAdV infection in the differential diagnosis for high-risk patients. Because half the patients reviewed had a co-infection by other viruses, the presence of other viral infections does not exclude the possibility of an HAdV infection.

The tools for diagnosing HAdV infections include a viral culture, viral antigen assay, histopathologic studies and PCR. The PCR is the best option both in terms of sensitivity and shorter turnaround time [33]. A high serum viral load (e.g., >1.3 × 10^7^ copies/ml) has been associated with poor outcomes in HSCT recipients [20]. In our review, the serum viral load ranged from 352 to more than 1.0 × 10^10^ copies/ml, although three patients survived despite viremia exceeding 1.0 × 10^7^ copies/ml. Weekly monitoring of the HAdV load in the peripheral blood using quantitative PCR is recommended for high-risk HSCT patients, but there is no consensus as to when treatment should begin [32,34]. Furthermore, quantitative PCR may not be readily available for weekly monitoring in certain communities, and alternative methods such as an antigen assay, might be insufficient to determine the true pathogenicity when HAdV is isolated from a nonsterile source such as a respiratory sample. It might be beneficial to use quantitative PCR for HAdV to detect respiratory pathogens in HSCT patients with respiratory symptoms as occurred for our patient.

Regarding treatment options, cidofovir and ribavirin are used in treating immunocompromised patients, and 77/80 (96.3%) patients reviewed received either cidofovir or ribavirin. Most of the evidence for efficacy against HAdV, however, is present for cidofovir and ribavirin has an activity only limited to species C [33]. The commonest side effect of cidofovir is nephrotoxicity, and close monitoring of the renal function is crucial in patients with a hematologic malignancy due to the frequent, concomitant use of other nephrotoxic agents. Due to the severe adverse effects associated with cidofovir, physicians may be reluctant to use the drug if patients do not have high-level viremia. This reluctance may delay the commencement of therapy for disseminated adenovirus infections. Ganciclovir, commonly used to treat CMV infections, has been suggested a possible treatment for HAdV infections [35]; however, there are currently no official recommendations. In our review, three patients received ganciclovir for the treatment of co-infections or as a prophylaxis against CMV infections [12,28], and one patient received the drug for the treatment of an HAdV infection [7]. Brincidofovir, an experimental oral lipid ester of cidofovir with less nephrotoxicity, is being tested as a possible treatment for HAdV infections (ClinicalTrials.gov Identifier: NCT02087306) [36] and has shown a favorable safety and tolerability profile in a recent study [37]. As the increased risk of HAdV infection is associated with T-cell depleted grafts and lymphocytopenia, it is crucial to reduce immunosuppressive treatment, including graft-versus-host disease prophylaxis, whenever possible [1]. Other immunotherapies, including donor lymphocyte infusion and donor-derived HAdV-specific T cells, are now a treatment option for patients with a HAdV infection that is recalcitrant to conventional antiviral therapy. In our review, one study reported a promising outcome for a combined use of cidofovir and low-dose donor lymphocyte infusion [19], and three studies examining the use of HAdV-specific T cells in a treatment regimen reported that two of six patients survived [15,16,21].

Although exogenous infection in the inpatient setting is a rather rare cause of HAdV-related infection [1], outbreaks of infection on hematology wards have been reported [38,39]. Since poor prognosis occurred in HSCT patients with a disseminated adenovirus infection, infection control such as restriction on visits might be beneficial to prevent exogenous infection by nosocomial acquisition in the inpatient setting.

## Conclusion & future perspective

We reported a case of disseminated adenovirus infection after CBT in a patient with a lymphoma which had a poor outcome. Our literature review also demonstrated a poor prognosis in HSCT patients with hematologic malignancies even with antiviral therapy. Although mortality rate in our review might be influenced by reporting bias since not all patients with a disseminated adenovirus infection have been reported, the disease is still notable for its fatal outcome. Because there are no clear guidelines for the diagnosis and treatment of HAdV infections in immunocompromised patients, a high index of suspicion for the presence of HAdV infection and pursuing diagnostic tests in a timely manner are crucial to improve outcomes.

Current recommendations for HAdV screening and monitoring in HSCT patients are still relatively diverse, and further studies are needed to provide the standardized approach in this area. Furthermore, the development of novel less toxic antiviral agents is expected to reduce the concern on side effect when using in either preemptive or targeted treatment. In addition, antiviral immunotherapy with HAdV-specific T cells might offer the potential for improvement in both the prevention and treatment of HAdV infections. We hope that the current review guides researchers and clinicians to further refine diagnosis and treatment for both research studies and clinical practice.

**Executive summary**

- A case of disseminated adenovirus infection in an adult patient with hematopoietic stem cell transplantation (HSCT) was described.
- Despite the use of an antiviral agent, the patient’s prognosis was poor.
- Disseminated adenovirus infection can occur in immunocompromised patients with HSCT.
- We performed a literature review of disseminated adenovirus infection with adult HSCT patients.
- The clinical manifestation of disseminated adenovirus infection varies; in our review, almost half of the cases had pneumonia.
- Although adenovirus load in the peripheral blood using quantitative PCR is helpful to make a diagnosis, the best timing of test remains uncertain.
- The treatment options include antiviral agents and immunotherapies such as donor lymphocyte infusion and donor-derived human adenovirus-specific T cells.
- Our review of the literature showed a high mortality rate of more than 70% in adult HSCT patients with disseminated adenovirus infection.
