# Supplementary material for: Disseminated adenovirus infection in a patient with a hematologic malignancy: a case report and literature review
Source: Future Sci OA. 2019 Aug 28;5(8):FSO412. doi: 10.2144/fsoa-2019-0072 (PMC6745594; doi:10.2144/fsoa-2019-0072)
Supplement: Supplementary file 1 [file fsoa-05-412-s1.docx]

**Supplementary Table 1.** Clinical characteristics of human adenovirus infection in hematopoietic stem cell transplant recipients

| No. | Age (yrs) /Sex | Type of HSCT | Timing of infection after HSCT | First symptoms | Infection site | Peak serum viral load or at diagnosis (copies/ml) | Antiviral treatment for HAdV | Presence of other viral co-infection | Outcome | Ref. |
| --- | --- | --- | --- | --- | --- | --- | --- | --- | --- | --- |
| 1 | 25/M | CBT | day 42 | cough, fever, hematuria | lung, kidney | > 1.0 × 10^6 | cidofovir | none | survived | 5 |
| 2 | 58/F | BMT | day 79 | fever, fatigue | liver | N/A | none | CMV, EBV | died | 6 |
| 3 | 35/F | BMT | day 24 | hematuria | lung, urinary tract, colon | 5.0 × 10^6 | none | none | survived | 7 |
| 4 | 39/M | PBSCT | day 35 | vomiting, diarrhea | liver, colon | 1.4 × 10^4 | cidofovir, ribavirin | none | died | 8 |
| 5 | 26/M | BMT, PBSCT | more than 6 years | fever, cough, diarrhea | lung, colon, liver | > 1.0 × 10^7 | cidofovir | none | survived | 9 |
| 6 | 62/M | PBSCT | day 28 | fever, erythroderma | urinary tract, liver | N/A | none | none | died | 10 |
| 7 | 20/M | BMT | day 10 | hematuria | urinary tract, bone marrow, eye, lung | N/A | ribavirin | none | died | 11 |
| 8 | 59/F | PBSCT | day 20 | diarrhea | lung, colon | 1.0 × 10^7 | cidofovir, brincidofovir | influenza A, RSV | survived | 12 |
| 9 | 54/F | HSCT | day 51 | shortness of breath, cough | lung, colon | 3.0 × 10^6 | brincidofovir | EBV, CMV | died | 12 |
| 10 | 34/M | HSCT | day 21 | fever, diarrhea | colon, liver, urinary tract | 2.4 × 10^5 | brincidofovir | CMV, BKV | survived | 12 |
| 11 | 48/M | HSCT | day 335 | N/A | lung, eye | 41,993 | cidofovir | CMV, HHV-6 | died | 12 |
| 12 | 44/F | HSCT | day 135 | N/A | lung, urinary tract, colon | 352 | cidofovir | CMV, EBV | survived | 12 |
| 13 | 22/M | HSCT | day 1,140 | N/A | lung, eye, colon | 972 | none | none | survived | 12 |
| 14 | 39/M | HSCT | day 237 | N/A | lung, urinary tract, colon | 165,415 | cidofovir | HSV-1, PIV-3 | died | 12 |
| 15 | 30/F | HSCT | day 20 | N/A | lung, colon | 3133 | none | CMV | survived | 12 |
| 16 | 28/M | HSCT | day 67 | N/A | colon, urinary tract | 1593 | cidofovir | EBV | survived | 12 |
| 17 | 51/F | HSCT | day 124 | malaise | liver | > 5.0 × 10^7 | none | none | died | 13 |
| 18 | 34/M | BMT | day 88 | diarrhea | lung, colon | 347,770 | cidofovir | CMV, HHV-6 | died | 14 |
| 19 | 48/F | BMT | day 371 | change in general condition | lung | 111,100 | cidofovir | none | died | 14 |
| 20 | 31/? | PBSCT | day 215 | N/A | colon | 8.71 × 10^4 | none | N/A | survived | 15 |
| 21 | 27/? | CBT | day 126 | N/A | colon | 8.77 × 10^5 | cidofovir | N/A | survived | 15 |
| 22 | 52/F | CBT | N/A | N/A | lung | N/A | cidofovir, ribavirin | CMV, JCV, BKV | died | 16 |
| 23 | 27/M | CBT | N/A | N/A | lung | N/A | cidofovir | CMV, BKV | died | 16 |
| 24 | 26/M | BMT | N/A | N/A | none | N/A | cidofovir, ribavirin | CMV, BKV, HSV | died | 16 |
| 25 | 49/M | PBSCT | N/A | N/A | lung | N/A | cidofovir, ribavirin | CMV, EBV | died | 16 |
| 26 | 23/F | PBSCT | N/A | N/A | lung | N/A | cidofovir, ribavirin | CMV, BKV | died | 16 |
| 27 | N/A | HSCT | day 53 | N/A | kidney, urinary tract | 3,981,072 | cidofovir, brincidofovir | N/A | died | 17 |
| 28 | N/A | HSCT | day 47 | N/A | kidney, urinary tract | 125,893 | cidofovir, brincidofovir | N/A | survived | 17 |
| 29 | N/A | HSCT | day 38 | N/A | colon | 10 × 10^7 | cidofovir, brincidofovir | N/A | died | 17 |
| 30 | N/A | HSCT | day 19 | N/A | colon | 3,981,072 | cidofovir, brincidofovir | N/A | died | 17 |
| 31 | N/A | HSCT | day 18 | N/A | lung | 7,943 | cidofovir | N/A | died | 17 |
| 32 | N/A | HSCT | day 14 | N/A | colon | 3,162,278 | brincidofovir | N/A | died | 17 |
| 33 | 47/M | PBSCT | day 12 | N/A | lung | 310,000 | cidofovir | none | survived | 18 |
| 34 | 59/F | BMT | day 31 | N/A | lung | 2,500,000 | cidofovir | CMV | died | 18 |
| 35 | 39/M | BMT | day 10 | N/A | lung, urinary tract | > 10^8 | cidofovir | none | died | 19 |
| 36 | 53/M | PBSCT | day 20 | N/A | lung, urinary tract | > 10^8 | none | none | died | 19 |
| 37 | 58/M | PBSCT | day 20 | N/A | lung, urinary tract | > 10^8 | none | none | died | 19 |
| 38 | 21/F | BMT | day 18 | N/A | urinary tract | > 10^8 | cidofovir | none | survived | 19 |
| 39 | 45/M | PBSCT | day 10 | N/A | liver, urinary tract | > 10^6 | cidofovir | none | survived | 19 |
| 40 | 55/M | PBSCT | day 5 | N/A | urinary tract | > 10^6 | cidofovir | CMV | survived | 19 |
| 41 | 58/M | PBSCT | day 10 | N/A | urinary tract | > 10^6 | cidofovir | none | survived | 19 |
| 42 | 39/M | HSCT | N/A | N/A | N/A | 1.8 × 10^9 | N/A | N/A | died | 20 |
| 43 | 41/M | HSCT | N/A | N/A | N/A | 2.0 × 10^4 | N/A | N/A | died | 20 |
| 44 | 49/M | HSCT | N/A | N/A | N/A | 7.0 × 10^5 | N/A | N/A | died | 20 |
| 45 | 34/M | HSCT | N/A | N/A | N/A | 4.0 × 10^4 | N/A | N/A | died | 20 |
| 46 | 54/M | HSCT | N/A | N/A | N/A | 4.4 × 10^4 | N/A | N/A | survived | 20 |
| 47 | 59/F | HSCT | N/A | N/A | N/A | 1.0 × 10^8 | N/A | N/A | died | 20 |
| 48 | 28/M | HSCT | N/A | N/A | N/A | 1.6 × 10^4 | N/A | N/A | died | 20 |
| 49 | 33/F | HSCT | N/A | N/A | N/A | 2.0 × 10^7 | N/A | N/A | died | 20 |
| 50 | 51/M | HSCT | N/A | N/A | N/A | 1.0 × 10^9 | N/A | N/A | died | 20 |
| 51 | 49/M | HSCT | N/A | N/A | N/A | 3.0 × 10^4 | N/A | N/A | died | 20 |
| 52 | 62/M | HSCT | N/A | N/A | N/A | 1.3 × 10^5 | N/A | N/A | survived | 20 |
| 53 | 68/M | HSCT | N/A | N/A | N/A | 1.7 × 10^4 | N/A | N/A | died | 20 |
| 54 | 39/M | HSCT | N/A | N/A | N/A | 6.0 × 10^4 | N/A | N/A | died | 20 |
| 55 | 47/F | HSCT | N/A | N/A | N/A | 3.0 × 10^5 | N/A | N/A | survived | 20 |
| 56 | 57/F | HSCT | N/A | N/A | N/A | 3.0 × 10^5 | N/A | N/A | died | 20 |
| 57 | 39/F | HSCT | N/A | N/A | N/A | 4.0 × 10^4 | N/A | N/A | survived | 20 |
| 58 | 27/M | HSCT | N/A | N/A | N/A | 4.0 × 10^6 | N/A | N/A | died | 20 |
| 59 | 38/F | HSCT | N/A | N/A | N/A | 5.2 × 10^7 | N/A | N/A | died | 20 |
| 60 | 54/F | HSCT | N/A | N/A | N/A | 5.2 × 10^6 | N/A | N/A | survived | 20 |
| 61 | 23/F | HSCT | N/A | N/A | N/A | 4.0 × 10^7 | N/A | N/A | died | 20 |
| 62 | 23/F | HSCT | N/A | N/A | N/A | 3.0 × 10^5 | N/A | N/A | died | 20 |
| 63 | 25/M | HSCT | N/A | N/A | N/A | 2.3 × 10^6 | N/A | N/A | survived | 20 |
| 64 | 50/M | HSCT | N/A | N/A | N/A | 5.0 × 10^5 | N/A | N/A | survived | 20 |
| 65 | 39/F | HSCT | N/A | N/A | N/A | 6.4 × 10^8 | N/A | N/A | died | 20 |
| 66 | 48/M | HSCT | N/A | N/A | N/A | 3.5 × 10^7 | N/A | N/A | died | 20 |
| 67 | 28/F | CBT | day 65 | N/A | N/A | > 10 × 10^7 | cidofovir | HSV | died | 21 |
| 68 | 28/F | CBT | day 197 | N/A | N/A | > 10 × 10^6 | cidofovir, ribavirin | HHV-6 | died | 21 |
| 69 | 30/M | CBT | day 88 | N/A | N/A | > 10 × 10^6 | cidofovir | none | died | 21 |
| 70 | 25/F | PBSCT | day 58 | N/A | N/A | N/A | cidofovir | EBV | died | 21 |
| 71 | 33/F | CBT | day 214 | N/A | N/A | > 10 × 10^6 | cidofovir | EBV, HHV-6 | died | 21 |
| 72 | 33/M | BMT | day 27 | N/A | N/A | N/A | cidofovir | EBV | died | 21 |
| 73 | 33/M | PBSCT | day 175 | N/A | N/A | N/A | none | HHV-6 | survived | 21 |
| 74 | 24/M | BMT | day 518 | N/A | N/A | N/A | none | none | died | 21 |
| 75 | 20/M | BMT | day -4 | N/A | N/A | N/A | cidofovir | CMV | died | 21 |
| 76 | 48/M | BMT | day 90 | N/A | N/A | N/A | cidofovir | none | die | 21 |
| 77 | 37/M | BMT | day 59 | N/A | N/A | N/A | cidofovir | CMV | died | 21 |
| 78 | 36/M | BMT | day 241 | N/A | N/A | N/A | cidofovir | EBV | died | 21 |
| 79 | 37/F | HSCT | day 36 | N/A | none | 19,953 | cidofovir | N/A | died | 22 |
| 80 | 53/M | HSCT | day 310 | N/A | urinary tract | 12,589 | cidofovir | N/A | died | 22 |
| 81 | 55/M | HSCT | day 63 | N/A | none | 3,981 | cidofovir | N/A | died | 22 |
| 82 | 48/M | HSCT | day 16 | N/A | colon | > 1.0 × 10^8 | cidofovir | N/A | died | 22 |
| 83 | 56/M | HSCT | day 60 | N/A | lung | 63,096 | cidofovir | N/A | died | 22 |
| 84 | 26/M | HSCT | day 240 | N/A | colon | 3,981 | cidofovir | N/A | died | 22 |
| 85 | 60/M | HSCT | day 250 | N/A | colon | 501 | cidofovir | N/A | died | 22 |
| 86 | 48/M | HSCT | day 20 | N/A | urinary tract | 3,981,072 | ribavirin | N/A | died | 22 |
| 87 | 31/F | HSCT | day 150 | N/A | colon | > 1.0 × 10^8 | ribavirin | N/A | died | 22 |
| 88 | 23/M | HSCT | day 240 | N/A | none | 501 | none | N/A | survived | 22 |
| 89 | 64/M | HSCT | day 180 | N/A | colon | 3,162 | cidofovir | N/A | died | 22 |
| 90 | 53/F | HSCT | day 110 | N/A | colon | 1,995 | cidofovir | N/A | died | 22 |
| 91 | 54/M | HSCT | day 160 | N/A | urinary tract | 25,119 | cidofovir | N/A | died | 22 |
| 92 | 20/? | BMT | day 167 | fever | lung, colon, CNS | 1,584,893 | cidofovir | none | died | 23 |
| 93 | 60/M | HSCT | day 148 | N/A | liver, colon | > 1.0 × 10^10 | cidofovir | N/A | died | 24 |
| 94 | 50/M | HSCT | day 19 | N/A | urinary tract | 1.0 × 10^8 | ribavirin | N/A | died | 25 |
| 95-112 | Median age 40  /M:16 F:2 | PBSCT: 7  BMT: 6  CBT: 5 | day 70 (range: 24–304 days) | N/A | intestine, bladder (n=5),  lung, bladder (n=4),  lung, intestine (n=3),  lung, intestine, bladder (n=3),  lung, bladder, kidney (n=1),  lung, liver (n=1)  lung, intestine, liver, bladder, kidney (n=1) | N/A | none: 10  cidofovir: 8 | N/A | died: 12  survived: 6 | 26 |
| 113-125 | N/A | BMT | N/A | N/A | lung: 5  no lung: 8 | N/A | iv ribavirin (n= 12) and/or aerosolized ribavirin (n= 4) | N/A | died: 8  survived: 5 | 27 |
| 126 | 50/M | BMT | day 20 | abdominal pain, hematuria | urinary tract, lung | not detected at diagnosis | none | none | died | 28 |
| 127 | 41/F | BMT | day 427 | N/A | lung, colon | N/A | none | none | died | 28 |
| 128 | 39/F | BMT | day 180 | diarrhea, abdominal pain, nausea, vomiting | colon | N/A | none | none | died | 29 |
| 129 | 20/M | HSCT | day 70 | abdominal pain, hematuria, vomiting | urinary tract | N/A | cidofovir | BKV | died | 30 |
| 130 | 46/M | CBT | day 11 | fever, cough | lung, CNS | 3.2 × 10^7 | cidofovir | none | died | current case |
